# In Situ Study and Improvement of the Temperature Increase and Isothermal Retention Stages in the Polyacrylonitrile (PAN) Fiber Pre-Oxidation Process

**DOI:** 10.3390/polym16040547

**Published:** 2024-02-18

**Authors:** Ye Cui, Lizhi Liu, Lixin Song, Sanxi Li, Ying Wang, Ying Shi, Yuanxia Wang

**Affiliations:** 1 Polymer High Functional Film Engineering Research Center of Liaoning Province, Shenyang University of Chemical Technology, Shenyang 110142, Chinalizhiliu@aliyun.com (L.L.);; 2 School of Materials Science and Engineering, Shenyang University of Technology, Shenyang 110870, China; 3 Research and Development, Dongguan HAILI Chemical Material Co., Ltd., Dongguan 523808, China

**Keywords:** PAN, temperature increase stage, isothermal retention stage, SAXS/WAXD, pre-oxidation process

## Abstract

The pre-oxidation process of Polyacrylonitrile (PAN) fibers is a complex procedure involving multiple stages of temperature increase and isothermal temperature retention. However, the impact of the temperature increase stage on PAN fiber has often been overlooked. To address this, samples were collected before and after the temperature increase and isothermal retention stages, treating them as separate influencing factors. Therefore, the pre-oxidation process can be divided into four distinct stages: (1) A temperature increase stage before the cyclization reactions: the PAN fiber’s small-size crystals melt, and the crystal orientation changes under fixed tension, leading to shrinkage and increased orientation of the micropore. (2) An isothermal retention stage before the cyclization reactions: The crystal structure maintains well, resulting in minimal micropore evolution. The PAN fiber’s crystal orientation and micropore orientation increased under fixed tension. (3) A temperature increase stage after the cyclization reactions: The PAN fiber’s crystal melts again, reducing the average chord length and relative volume of the micropore. However, the PAN fiber can recrystallize under fixed tension. (4) An isothermal retention stage after the cyclization reactions: Significant crystal melting of the PAN fiber occurs, but the highly oriented crystals are maintained well. The average chord length and relative volume of the micropore increase. Recommendations for improving the pre-oxidation process are made according to these stages.

## 1. Introduction

PAN fibers have a high melting point and exhibit molecular chain alignment along the fiber axis owing to stretching during the preparation process [1]. This structure promotes cyclization reactions between molecular chains at low temperatures, resulting in the formation of ladder molecular structures [2]. The ladder structure remains essentially intact during high-temperature carbonization, leading to a high carbon yield [3]. This makes PAN fibers suitable for producing products with excellent mechanical properties. The pre-oxidation process of Polyacrylonitrile (PAN) fibers is a complex procedure involving multiple stages of temperature increase and isothermal temperature retention [4,5,6,7]. This is because it reduces the occurrence of concentrated heat release in PAN fibers [8], thus minimizing the formation of structural defects [9]. Multiple increasing temperatures and isothermal retention stages result in the pre-oxidation process consuming more time and energy [10], ultimately increasing the manufacturing cost of carbon fibers. Therefore, much research has been conducted on the pre-oxidation process of PAN fibers to reduce the pre-oxidation process cost and enhance the mechanical properties of carbon fibers.

Research on improving the pre-oxidation process of PAN can be divided into two aspects. One involves changing the processing conditions during the pre-oxidation stage, such as modifying the stretching [11,12], altering the pre-oxidation retention time [4,7], or adjusting the pre-oxidation temperature [13]. The primary focus affects the crystal structure of PAN fibers during the pre-oxidation process, resulting in an improvement in the microstructure and mechanical properties of carbon fibers. The second aspect involves physical or chemical pretreatment methods for the original silk, such as annealing prior to the pre-oxidation process [14,15], treatment with hydroxylamine hydrochloride [16], heating in a nitrogen atmosphere [9,17], or microwave treatment [18]. These modifications facilitate the efficiency of cyclization reactions during the pre-oxidation process, saving time and energy, enhancing carbon yield, and increasing the mechanical properties of carbon fibers.

Although previous studies investigated the temperature [19,20] and retention time [21] during the pre-oxidation process, these studies have yet to fully understand the impact of temperature or retention time on the pre-oxidation process. This is because previous researchers did not distinguish between the temperature increase and isothermal retention stages. Whether studying the impact of pre-oxidation temperature or retention time on PAN fiber structure, the sampling was conducted after the fiber underwent the isothermal retention temperature stage. While this approach ensures complete cyclization reactions at the specified temperature, it overlooks the influence of temperature increase stages on PAN fiber structure. However, in the pre-oxidation process, the temperature range is wide, and the heating process is also complex, involving multiple temperature increase stages. Therefore, the influence of the temperature increase stage on PAN fiber structure cannot be ignored.

In this study, Synchrotron Wide-Angle X-ray Diffraction (WAXD) and Synchrotron Small Angle X-ray Scattering (SAXS) were employed to investigate the in situ pre-oxidation process of PAN fibers. Samples were taken before and after the heating and isothermal retention stages by separating the temperature increase and isothermal retention stages as distinct influencing factors within the pre-oxidation process. The evolution of the microstructures of PAN fibers during the temperature increase and isothermal retention stages was examined. Considering the influence of fixed tension on the structure of PAN fibers during the pre-oxidation process [22], the PAN fiber WAXD/SAXS experiments were conducted at fixed conditions. Additionally, an analysis of carbon fiber structures with different mechanical properties was employed to understand the microstructure associated with excellent mechanical performance. Based on these findings, and combined with the characteristics of the microstructure during the temperature increase and isothermal retention stages, recommendations for improving the pre-oxidation process are proposed.

## 2. Experimental Section

### 2.1. Sample Information

The PAN fiber was obtained from China Petrochemical Corporation. It involved copolymerization of acrylonitrile (AN), itaconic acid (IA), and methyl acrylate (MA) in a solution using dimethyl sulfoxide as a solvent and azobisisobutyronitrile as an initiator. The monomer mass ratio of AN:IA: MA was 98:1:1, and the molecular weight was 1.5 × 10^5^ g/mol.

The preparation of PAN precursor fibers for carbon fiber production requires high orientation and crystallinity. To achieve high orientation and crystallinity in PAN fiber, it must undergo multiple stages of stretching rather than just a single stretch. Therefore, it is necessary to carry out a Coagulation draft, Water bath draft, and Vapor bath draft.

**Figure 1:**
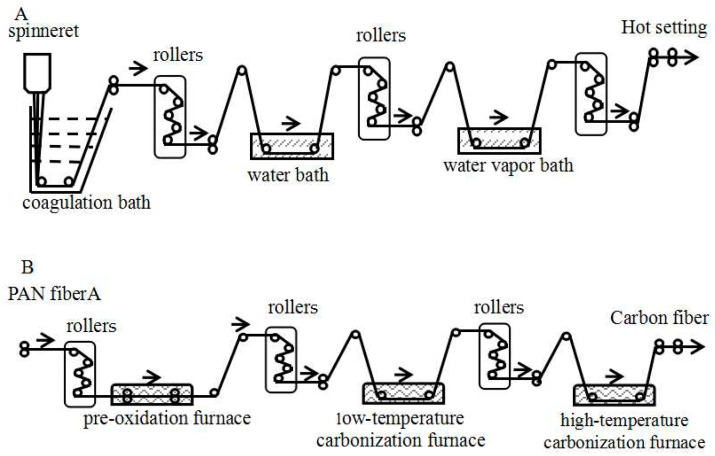
Schematic diagram of PAN fiber A preparation (**A**) and carbon fiber preparation (**B**).

Figure 1A describes the wet spin process of PAN fiber A, where the PAN fiber was used in multiple stages of stretching, washing, and drying [23,24]. First, the spinning dope was passed through a spinneret into a mixture of dimethyl sulfoxide and water (DMSO/H_2_O) using the DMSO/H_2_O mixture as a coagulation bath. The coagulated PAN fiber was subsequently washed and stretched in a water bath with rollers. Afterward, the wet PAN fibers underwent treatment with high-temperature water vapor and stretching using a water vapor roller unit. This process served to remove water and collapse any voids within the fibers. Finally, the PAN fiber was obtained after a hot setting.

Figure 1B illustrates the carbon fiber preparation process, which consisted of three stages: pre-oxidation, low-temperature carbonization, and high-temperature carbonization. Initially, the PAN fiber underwent pre-oxidation in an air atmosphere, forming a thermally stable ladder structure. The fiber then enters a low-temperature carbonization furnace with a nitrogen atmosphere, where cross-linking occurs, releasing small gaseous molecules and removing non-carbon elements. The ladder structure aids in removing nitrogen, facilitates cross-linking, and transforms the linear chain into a planar structure. Finally, in the high-temperature carbonization stage, molecular chains rearrange, further developing the planar structure and forming a disordered graphite structure layer.

The Toary T800 was purchased from Toray Industries Inc., Tokyo, Japan. The samples were obtained from the actual carbon fiber industrial line using the previously described spinning process. Sample processing conditions are detailed in Table 1, Table 2 and Table 3.

**Figure 2:**
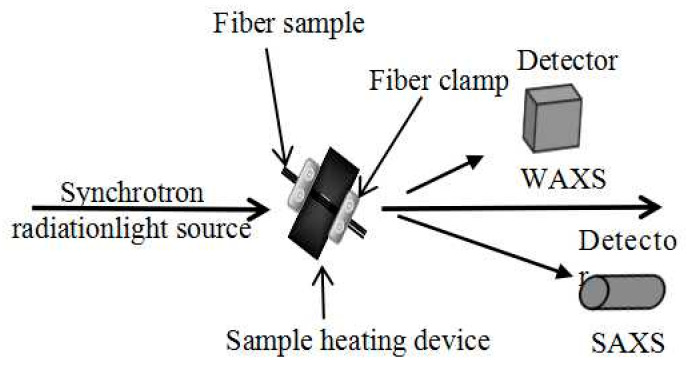
Schematic diagram of PAN fiber in situ SAXS/WAXD analysis.

In order to accurately distinguish between the temperature increase and isothermal retention stages, this study utilized in situ SAXS/WAXD analysis to evaluate changes in PAN fiber structures during the pre-oxidation process. This approach mitigates the potential impact of temperature drops on the structure of PAN fibers during the sample selection process. In this research, the PAN fiber was fixed using a fiber clamp and subjected to multiple heating steps, as depicted in Figure 2. Initially, the PAN fiber was heated to 180 °C at a rate of 30 °C/min and isothermally maintained at this temperature for 15 min. Subsequently, it underwent heating to 220 °C and was again isothermally maintained for 15 min, followed by heating to 240 °C at a rate of 30 °C/min and isothermally maintained for 15 min, ultimately reaching 270 °C and being isothermally maintained for a further 15 min. The pre-oxidation process involved multiple heating cycles of the PAN fiber in an air atmosphere. Therefore, the specific parameters used in this study are provided in Table 3, while low/high-temperature carbonization does not require multiple heating cycles.

### 2.2. Thermal Analysis

**Figure 3:**
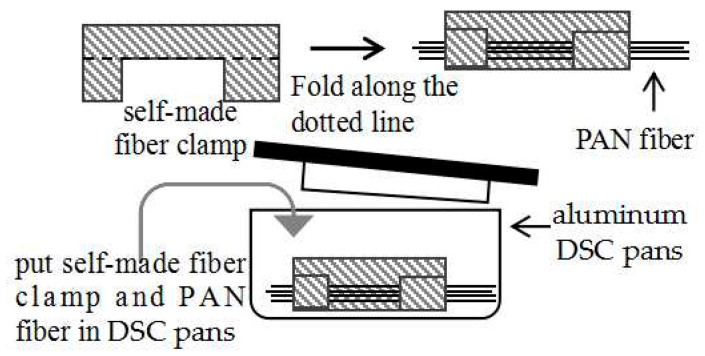
Schematic diagram of the fiber fixing method in the aluminum DSC pans.

To prevent fiber shrinkage during the pre-oxidation process, it is crucial to apply fixation tension. The presence of fixation tension has a significant impact on the process [22]. Thus, to mimic actual production conditions, Differential Scanning Calorimetry (DSC) measurements were conducted on the fibers in a stretched state. This was achieved by fixing the fibers using a self-made fiber clamp and holding tension throughout the experiment, as illustrated in Figure 3. In this study, iron was utilized as the material for constructing the self-made fiber clamp. An equal mass of patches was also placed in the reference crucible to eliminate the influence of the fixed clamp on the data.

Approximately 5 mg of PAN fiber was placed in standard aluminum DSC pans while holding tension. The DSC measurements were carried out using the Q100 DSC from TA Instruments Co., New Castle, DE, USA. The sample was heated to 350 °C at a rate of 10 °C/min and then cooled to 40 °C at the same rate. N_2_ atmosphere was used with a flow rate of 50 mL/min.

### 2.3. Synchrotron Wide-Angle X-ray Diffraction (WAXD)

Synchrotron WAXD experiments were performed on Beamline BL16B1 at the Shanghai Synchrotron Radiation Facility (SSRF), Shanghai, China. The storage ring was operated at 3.5 GeV and 300 mA. The wavelength (λ) employed was 0.124 nm. A three-slit system was used to define the incident beam. The sample-to-detector distance for the WAXD analysis was 265 mm. The collected WAXD images were calibrated using a silver behenate standard. Scattering intensity was detected by a two-dimensional (2D) Pilatus3 2M X-ray detector from DECTRIS Ltd., Baden, Switzerland, with a pixel size of 172 µm × 172 µm. The detector resolution was 1475 × 1679 pixels, and the collection time for each image was 60 s. Background scattering and beam intensity fluctuations were corrected for all measured patterns. The fiber bundles were well aligned throughout the analyses. The collected data were analyzed using the Fit2D software from ESRF (European Synchrotron Radiation Facility), Grenoble, France.

### 2.4. Synchrotron Small-Angle X-ray Scattering (SAXS)

Synchrotron SAXS experiments were performed on Beamline 1W2A at the Beijing Synchrotron Radiation Facility (BSRF), Beijing, China. The storage ring was operated at 2.5 GeV and 300 mA. The wavelength (λ) employed was 0.1542 nm. A three-slit system was used to define the incident beam. The sample-to-detector distance for the SAXS analysis was 1533 mm. The collected SAXS images were calibrated using mesoporous molecular sieves (SBA-15). Scattering intensity was detected by a two-dimensional (2D) MAR165 CCD X-ray detector from Mar USA Co., Palm Beach, FL, USA, with a pixel size of 80 µm × 80 µm. The detector resolution was 2048 × 2048 pixels, and the collection time for each image was 60 s. Background scattering and beam intensity fluctuations were corrected on the measured pattern. The fiber bundles were well aligned in the analysis. The collected data were analyzed using the Xpolar software, version 1.6.3.0, from Precision Works NY, Inc., Port Washington, DC, USA.

## 3. Results and Discussion

### 3.1. Thermal Analysis of PAN Fiber

The pre-oxidation process of PAN fibers is a complex procedure involving both physical structure changes and intricate chemical reactions [25,26,27]. As the temperature rises, the -CN groups in PAN fibers break and react with cyanide groups within or between molecular chains. This results in cross-linking and transforms the linear molecular configuration of PAN into a heat-resistant ladder structure [28,29], releasing a significant amount of heat. Hence, DSC analysis can be employed to evaluate the degree of pre-oxidation of PAN fibers based on the heat release.

**Figure 4:**
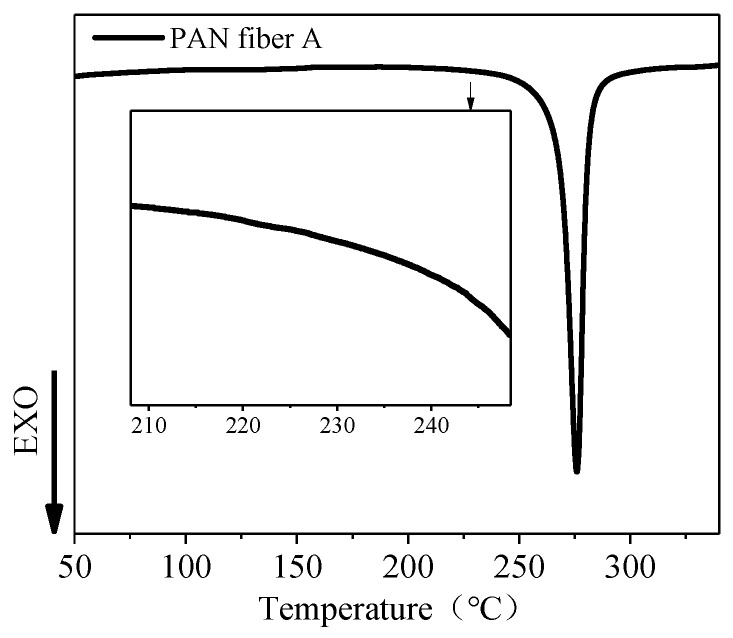
DSC thermograms of PAN fiber A heated at 10 °C/min in nitrogen.

The DSC curves in Figure 4 present the results of the heating process at a rate of 10 °C/min under a nitrogen atmosphere. The exothermic peak corresponds to the PAN fiber’s cyclization reaction that generated a thermally stable ladder structure with heat release [26,27]. Firstly, the exothermic peak of PAN fiber A began at around 220 °C, indicating the initiation of the cyclization reaction. Subsequently, as the temperature increased, the heat release rate of the cyclization reaction also increased, suggesting an acceleration in the cyclization reaction rate. The exothermic peak was observed at approximately 275 °C, representing the maximum rate of the cyclization reaction. However, as the temperature increased, the rate of heat release from cyclization decreased. Finally, the exothermic reaction of PAN fiber A concluded at nearly 290 °C, indicating complete cyclization.

### 3.2. Data Analysis

#### 3.2.1. WAXD Analysis

The chain crystalline orientation (*f*) was calculated using the Hermans orientation function [30]:

(1) $$f_{hkl,z}=\frac{3\langle {cos}^{2}\theta_{hkl,z}\rangle -1}{2}$$

where θ represents the angle between the chain axis and a reference axis, and <cos^2^ θ> is defined as:

(2) cos2θhkl=∫0π2Iθcos2θhklsinθhkldθhkl∫0π2Iθsin⁡θhkldθhkl

Here, I(θ) denotes the scattered intensity at an angle of θ. In this study, the fiber direction was considered the reference direction, and ƒ denotes the degree of crystal orientation of the 100 plane along the fiber direction. A value of 1 indicates perfect orientation, 0 indicates random orientation, and −0.5 denotes perfectly perpendicular orientation.

The crystallite size (L) was estimated using the Scherrer equation [31]:

(3) $$L=\frac{0.89\lambda }{\beta \cos\theta }$$

where *λ*, *θ*, and *β* denote the wavelength, diffraction angle, and width (in radians) at half-maximum intensity, respectively. For each hkl reflection, the value of L can be interpreted as the average crystal dimension perpendicular to the respective reflecting plane.

#### 3.2.2. Research on the Crystal Structure of Carbon Fiber

In addition to understanding the effects of the temperature increase and isothermal retention stages on the structure of PAN fibers during the pre-oxidation process, this study aimed to enhance the pre-oxidation technique to produce carbon fibers with excellent mechanical properties. Therefore, it was crucial to comprehend the structural characteristics associated with high-performance carbon fibers. Based on these characteristics, we assessed the structural changes during the temperature increase and isothermal retention stages of the pre-oxidation process. This study used Toray T800, which has a tensile strength of 5.9 GPa, and Carbon Fiber A, with a tensile strength of 4.9 GPa, as examples and compared the structures of these two carbon fibers to gain insights into the distinctive structural features of the high-performance carbon fiber T800. In order to eliminate the influence of fiber diameter on the structure of carbon fibers [32], the average diameter of the carbon fibers was measured, and both types of carbon fibers had a diameter of 5 μm. By comparing these two types of carbon fibers, we aimed to understand the structural characteristics that contribute to the excellent mechanical properties of carbon fibers. Based on these structural features, we provide appropriate suggestions for improvement of the pre-oxidation process.

**Figure 5:**
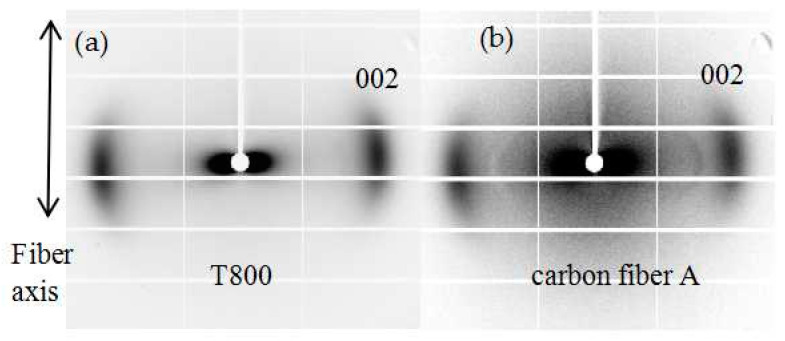
2D diffraction patterns of different carbon fibers at room temperature: (**a**) T800 carbon fiber, and (**b**) carbon fiber A prepared from PAN fiber.

The 2D WAXD patterns of the T800 carbon fiber and Carbon Fiber A are depicted in Figure 5. Both patterns display a distinct diffraction arc perpendicular to the fiber axis, corresponding to the reflection of the (002) crystal plane. These short diffraction arcs signify a high degree of fiber axis orientation in the carbon fiber crystals.

**Figure 6:**
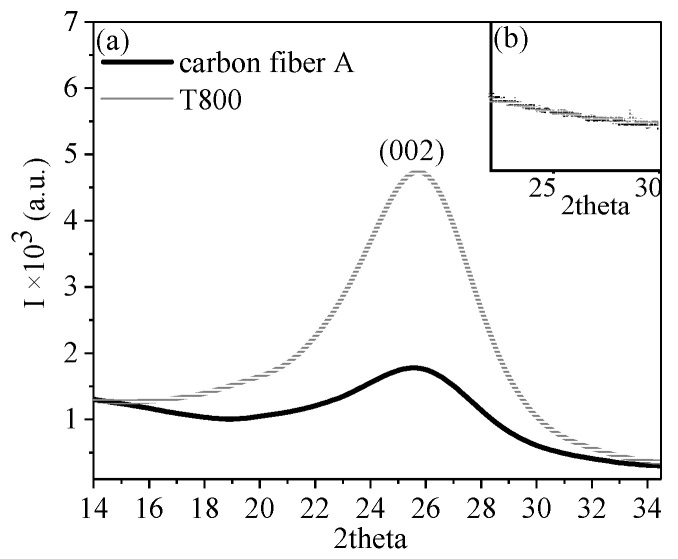
Linear WAXD profiles of carbon fibers along the fiber axis directions (**a**) and perpendicular to fiber axis directions (**b**) for a 10° integral range. Carbon fiber A was prepared from PAN fiber A (gray line) and T800 carbon fiber (black line).

The WAXD intensity profiles of carbon fibers obtained from the fiber axis directions and perpendicular to the fiber axis directions within a 10° integral range after normalization are depicted in Figure 6. Figure 6a exhibits a strong diffraction peak at 25° in both carbon fibers, indicating reflections from the (002) crystal plane. In contrast, Figure 6b exhibits no noticeable diffraction peaks, indicating the absence of crystal diffraction along the fiber axis direction. These findings suggest that the diffraction signals were primarily concentrated in the direction perpendicular to the fiber axis, indicating a high degree of crystal orientation along the fiber axis direction in the carbon fibers. Additionally, the intensity of the diffraction peak for T800 was significantly larger than that of carbon fiber A, suggesting that T800 had a higher crystallinity than carbon fiber A [20].

The crystal parameters of T800 and carbon fiber A were calculated using two-dimensional diffraction patterns and one-dimensional diffraction curves, as shown in Table 4. According to Table 4, both T800 and carbon fiber A exhibit similar crystal sizes of approximately 1.6 nm. However, the Hermans orientation of the T800 carbon fiber is greater than that of carbon fiber A. This can be attributed to a higher molecular chain orientation along the fiber axis directions and the regular parallel alignment of chain segments with the fiber axis directions in the T800 carbon fiber. Thus, it can be inferred that high-performance carbon fibers exhibit higher orientation and larger crystallinity.

#### 3.2.3. SAXS Analysis

The micropore structure of carbon fiber was analyzed using the Ruland [28] theoretical model and calculation of relevant micropore parameters such as the orientation distribution parameter (B_eq_), the length of the micropores (L), average chord length (L_P_), aspect ratio (L/L_P_), and the relative micropore volume (V_rel_).

According to Ruland’s relevant theoretical model [28], the micropores in the PAN fiber and carbon fiber are dispersed in a dilute system, and the micropore shape is cylindrical. The scattering intensity for the micropores can be defined as

(4) $$I_{V}\left(S\right)=I_{V}\left(S_{12},\;\;S_{3}\right)=\rho_{m}^{2}|\Phi_{D}{|}^{2}(S_{12})|\Phi_{L}{|}^{2}(S_{3})$$

where ρ_m_ is the electron density of the fiber in which the micropores are embedded, |Φ_D_|(S_12_) is the 2D Fourier transform of the shape function of the cross-section, and |Φ_D_|(S_12_) is the 1D Fourier transform of the shape function of the length. S_12_ and S_3_ are the components of the reciprocal space vector S in the directions perpendicular and parallel to the principal axis of the micro-void, respectively.

The width distribution of scattering intensity in the equatorial direction is defined as:

(5) $$B_{\pi /2}\left(S\right)=\frac{1}{I(S,\pi /2)}\int I(S,\emptyset )d\emptyset$$

**Figure 7:**
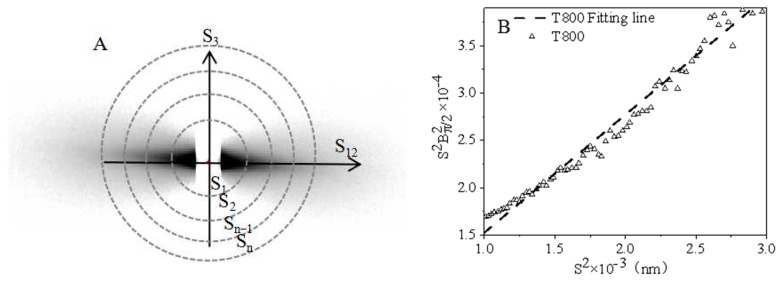
Schematic diagram of intensity integration along the azimuth (**A**) and S^2^~$S^{2}B_{\pi /2}^{2}$(S) plot of T800 carbon fiber (**B**).

The calculation method for B_π/2_(S) is shown in Figure 7A. Taking the scattering vector S_1_, S_2_, … S_n_ as the radius, the intensity of the scattering pattern is integrated along the azimuth. The integration result is derived from the diffraction intensity I (S_1,_ π/2), I (S_2,_ π/2)…I (S_n,_ π/2) to obtain the corresponding B_π/2_(S).

If the distribution of *φ* can be approximated using Gaussian distribution, the following relationship can be obtained:

(6) $$S^{2}B_{\pi /2}^{2}\left(S\right)=1/L^{2}+S^{2}B_{eq}^{2}$$

B_eq_ is the degree of micropore orientation away from the fiber axis, and L is the length of the micropores. The micropore length (L) and orientation angle (B_eq_) were obtained by linear fitting of the S^2^~$S^{2}B_{\pi /2}^{2}$(S) plot, as shown in Figure 7B.

The average chord length and relative micropore volume were obtained by taking the intensity values of medium and high angles into Equations (7) and (8).

(7) $$I(S,\pi /2)\propto n\rho_{m}\frac{L^{2}}{\sqrt{L+{\left(SLB_{eq}\right)}^{2}}}\frac{L_{p}^{4}}{{\left[1+{\left(2\pi L_{p}\right)}^{2}\right]}^{3/2}}$$

(8) $$V_{rel}\propto nLL_{p}^{2}$$

#### 3.2.4. Research on the Microporous Structure of Carbon Fiber

**Figure 8:**
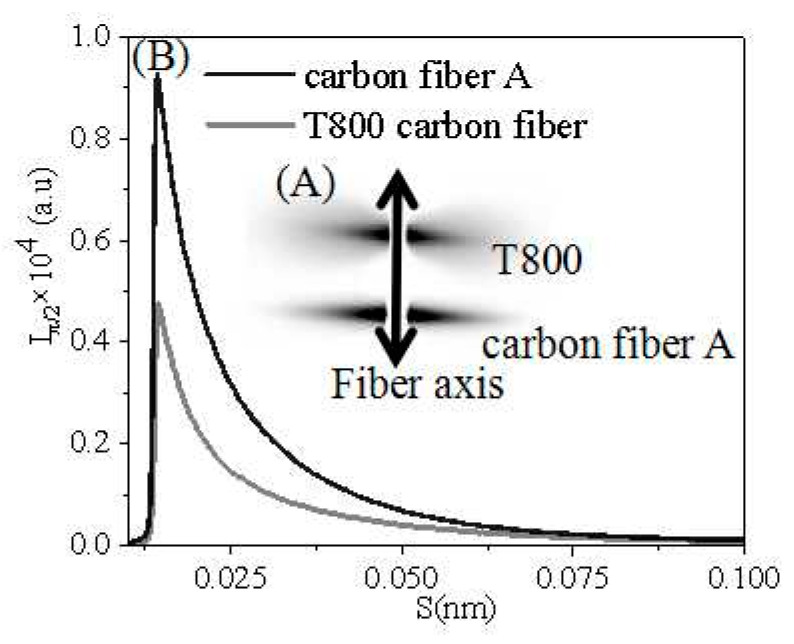
2D SASX patterns at room temperature (**A**); Linear SAXS Profiles of Toray T800 carbon fiber (black line) and carbon fiber A prepared from PAN fiber (gray line) in the transverse directions (**B**), where s = |s| = (2 sin θ)/λ, θ is the Bragg angle, and λ is the wavelength.

Micropore morphology of carbon fibers also plays a critical role in affecting mechanical properties, based on Griffith’s theory [33,34,35]. Hence, a two-dimensional small-angle X-ray scattering (2D SAXS) technique was employed to characterize the microporous structure of T800 and carbon fiber A to understand the structural characteristics of high-performance carbon fibers. The SAXS patterns of both carbon fibers are shown in Figure 8A, displaying a diamond-shaped pattern. This indicates a high degree of orientation in the micropores of both carbon fibers.

The SAXS profiles of two carbon fibers with a 2° range perpendicular to the fiber axis are presented in Figure 8B. The absence of a scattering peak in the figure indicates the lack of a periodic structure in all carbon fibers. The micropore parameters of T800 and carbon fiber A were calculated using two-dimensional diffraction patterns and linear diffraction curves, as shown in Table 4. Table 4 shows that the micropore length of T800 was significantly greater than that of carbon fiber A, while the average chord length (L_p_) was considerably smaller than that of carbon fiber A. This results in T800 carbon fibers possessing an elongated microporous structure. Such a structural characteristic increased the cross-sectional area experiencing load in the carbon fiber, consequently significantly enhancing its mechanical strength [36].

### 3.3. In Situ Study of the Pre-Oxidation Process

#### 3.3.1. Research on the Crystal Structure of PAN Fiber

In addition to the temperature increase stage, a prolonged isothermal retention stage is required in the pre-oxidation process. To distinguish between these stages and understand the structural changes of PAN fiber at different stages, this study employed in situ SAXS/WAXD analysis to investigate the pre-oxidation process. Data were collected before and after every temperature increase and isothermal retention stage to discriminate between the temperature increase and the isothermal retention stages, enabling a better understanding of the structural developments of PAN fibers at different stages.

**Figure 9:**
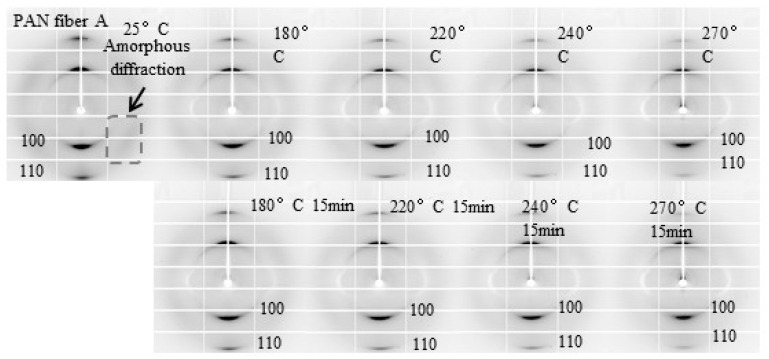
2D diffraction patterns of PAN fiber A during in situ study of the pre-oxidation process.

The two-dimensional diffraction pattern of PAN fiber A during the pre-oxidation process is shown in Figure 9. It can be observed from the figure that two distinct short diffraction arcs are evident in the pre-oxidation process of PAN fiber A, corresponding to the 100 and 110 crystal planes. The diffraction signal of the amorphous region can be observed at a 45° angle along the fiber axis. The figure shows that throughout the pre-oxidation process, the crystal diffraction signals of PAN fiber A consistently exhibit short arcs, indicating a high degree of orientation along the fiber axis [37,38,39,40]. As the temperature increases from 25 °C to 180 °C at a rate of 30 °C/min, the diffraction signal of the amorphous region disappears, showing that the amorphous region disappeared. After the isothermal retention stage at 180 °C, there is no significant change in the intensity of the diffraction peaks, suggesting that the isothermal retention stage at 180 °C had a relatively minor impact on the crystal structure of PAN fiber A. When the temperature reached 270 °C, the diffraction signal of the 110 crystal plane noticeably weakened, indicating the crystal structure of PAN fiber A was significantly destroyed.

**Figure 10:**
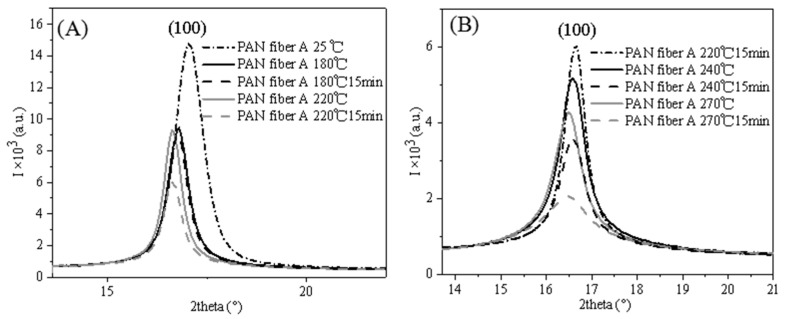
Linear WAXD profiles of PAN fiber A during in situ study of the pre-oxidation process in the transverse directions (integral range 10°): (**A**) The pre-oxidation process of PAN fiber A at the temperature increase stage from 25 °C to the isothermal retention stages of 220 °C; (**B**) The pre-oxidation process of PAN fiber A at the isothermal retention stages of 220 °C to the isothermal retention stages of 270 °C.

The WAXD profiles of PAN fiber A during the pre-oxidation process, obtained from the directions perpendicular to the fiber axis within a 10° integral range after normalization, are depicted in Figure 10. When the temperature increased from 25 °C to 180 °C, the diffraction peak intensity of the 100 crystal plane of PAN fiber A noticeably decreased, indicating a significant reduction in the fiber’s crystallinity [20]. According to the DSC data, no cyclization reaction occurred at this temperature. Hence, the decreased crystallinity was attributed to melting of the crystals [41]. Subsequently, after an isothermal retention temperature of 180 °C for 15 min, the diffraction peak intensity of the 100 crystal plane slightly decreased, suggesting a minor reduction in crystallinity and indicating that there was no significant melt of the crystals. After further increasing the temperature to 220 °C, the diffraction peak intensity of the 100 crystal plane slightly increased, resulting in a slight enhancement in crystallinity due to crystallization of the amorphous region molecular chains under fixed tension [6]. However, after the isothermal retention stage at 220 °C, the diffraction peak intensity of the 100 crystal plane once again significantly decreased, indicating a reduction in the crystallinity of PAN fiber A. Based on the DSC data, a cyclization reaction occurred at 220 °C. Therefore, the reduction in crystallinity during the isothermal retention stage was related to the cyclization reaction. After raising the temperature to 240 °C, the diffraction peak intensity of the 100 crystal plane declined again, indicating further melting of the crystals. After the isothermal retention stage at 240 °C, the diffraction peak intensity of the 100 crystal plane decreased once more, demonstrating a further reduction in the crystallinity of PAN fiber A. After increasing the temperature to 270 °C, the diffraction peak intensity of the 100 crystal plane increased again. Under the influence of fixed tension, the crystals of PAN fiber A that had not participated in the cyclization reaction were recrystallized, thereby enhancing the crystallinity of PAN fiber A. After the isothermal retention stage at 270 °C, the intensity of the 100 crystal plane diffraction peak noticeably declined, indicating a significant reduction in the crystallinity of PAN fiber A.

Considering the effects of the temperature increase and the isothermal retention stages as separate influencing factors, we observed a decrease in the crystallinity of PAN fiber A during the temperature increase stage (T < 220 °C), indicating melting of the PAN fiber crystals. During the isothermal retention stage, there was a slight reduction in crystallinity, suggesting only a small amount of melting occurred. After the cyclization reaction (T ≥ 220 °C), the temperature increase stage caused a decrease or an increase in crystallinity. This is because the PAN fiber molecular chains still possess strong mobility, allowing for recrystallization under fixed tension [42,43]. The results indicate that during the temperature increase stage, the primary occurrence is the melting of PAN fiber crystals, with relatively fewer cyclization reactions taking place to maintain the relatively strong mobility of PAN molecular chains. During the isothermal retention stage, only a reduction in crystallinity occurs. This is because cyclization reactions between or within the PAN fiber molecular chains lead to a decrease in the mobility of the PAN fiber molecular chains, rendering them unable to recrystallize even under the influence of fixed tension [11,44]. Therefore, during the isothermal retention stage, not only does melting of the crystals occur, but a significant amount of cyclization reactions also take place, further reducing the mobility of the PAN fiber molecular chains. Furthermore, during the temperature increase stage, the diffraction peak of the 100 crystal plane shifted toward smaller angles, indicating an increase in interplanar spacing. However, during the isothermal retention stage, the PAN fiber A peak position remained almost unchanged, showing no significant change in interplanar spacing. This suggests that changes in interplanar spacing are only influenced by temperature.

The crystal structural parameters of PAN fiber A, based on Figure 9 and Figure 10, are listed in Table 5. Table 5 shows that a substantial rise in crystal size and orientation occurred during the temperature increase stage from 25 °C to 180 °C before the cyclization reaction. However, as depicted in Figure 10 crystallinity declined in this stage, indicating the melting of small-sized, low-oriented crystals that caused the increase in crystal size and orientation. After the isothermal retention stage at 180 °C, there was a slight increase in crystal size, a slight decrease in crystallinity, and an increase in crystal orientation. This can be attributed to a few small-sized crystals that did not melt despite absorbing heat during the temperature increase stage from 25 °C to 180 °C. No cyclization reaction occurred during this stage, so the PAN chains maintained relatively strong mobility. Therefore, the fixed tension further enhanced the crystal orientation of the PAN fibers. As the temperature increased from 180 °C to 220 °C, there was a slight increase in crystal size and crystallinity, accompanied by a decrease in crystal orientation. This was due to the recrystallization of PAN fiber A under the influence of fixed tension, which increased crystal size and crystallinity. However, the crystal orientation did not increase but rather decreased. This is because the molecular mobility of the PAN fibers was improved at this stage, causing a reduction in crystal orientation. At the isothermal retention stages of 220 °C, 240 °C, and 270 °C, the crystal size and crystallinity decreased while the crystal orientation increased, and the interplanar spacing remained relatively constant. Those were associated with the gradual melting and cyclization reaction of large crystals. During the temperature increase stage, the crystal size and orientation continued to decrease, caused by the gradual melting of large crystals. Additionally, as the crystals absorb thermal energy, the ordered molecules oscillate more significantly around their lattice locations, which is reflected by a slight shift in the diffraction peak position toward a smaller angle [45].

The effects of the temperature increase and isothermal retention stages were considered separate influencing factors. After the cyclization reaction (T ≥ 220 °C), it was observed that the crystal orientation of PAN fibers decreased during all the temperature increase stages, while it increased during the isothermal retention stages. This can be attributed to the fact that during the temperature increase stage, the molecular chains of PAN fibers, which participate in the cyclization reaction, are relatively fewer. As a result, these molecular chains exhibit higher mobility. With increasing temperature, the molecular thermal motion intensifies, leading to a decrease in the orientation of large-sized, highly oriented crystals. On the other hand, the cyclization reaction primarily occurs during the isothermal retention stages, which decreases the mobility of PAN fiber molecular chains and enables the large-sized, highly oriented crystals that did not participate in the cyclization reaction to maintain their orientation.

**Figure 11:**
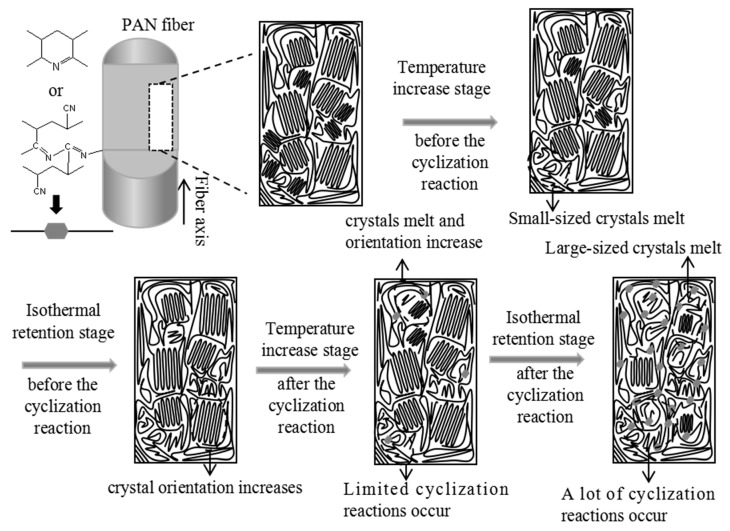
The crystal structure model of the PAN fiber during the pre-oxidation process.

The evolution of the crystal structure during the pre-oxidation process of PAN fibers is shown in Figure 11. The temperature increase and isothermal retention stages are considered separate phases. Based on the evolution of crystal structure parameters (crystallinity, crystal orientation, interplanar spacing, and crystal size), the entire pre-oxidation process can be divided into four stages: a temperature increase stage, an isothermal retention stage before cyclization reaction, a temperature increase stage, and an isothermal retention stage after the cyclization reaction.

Temperature increase stage before the cyclization reaction: Small-sized crystals in the PAN fiber melt upon absorbing heat, resulting in enhanced mobility of PAN molecular chains. The degree of crystal orientation increases under the influence of fixed tension.

Isothermal retention stage before the cyclization reaction: The melting of PAN fibers almost ceases, except for a few small-sized crystals that did not melt after absorbing heat during the temperature increase stage before the cyclization reaction occurs. At this stage, no cyclization reaction occurs within the PAN fibers, allowing the PAN chains to maintain a relatively strong mobility. Under the influence of fixed tension, the crystal orientations of PAN fibers are increased.

Temperature increase stage after the cyclization reaction: After absorbing heat, the crystals of the PAN fiber undergo melting and transform into an amorphous region. The majority of the heat is absorbed during the crystal melting process, which improves the mobility of the PAN molecular chains. At the same time, only some of the heat is absorbed by the PAN molecular chains in the amorphous region, which causes the cyclization reactions [28]. As a result, the PAN fiber maintains relatively high mobility, allowing for recrystallization under the influence of fixed tension.

Isothermal retention stage after the cyclization reaction: In contrast to the isothermal retention stage before the cyclization reaction, there is noticeable melting of the crystal that did not melt during the temperature increase stage owing to the heat released from the cyclization reaction of the PAN fiber molecular chains. Furthermore, the isothermal retention stage takes a long time, leading to many PAN molecular chains undergoing cyclization, resulting in decreased mobility of PAN molecular chains. Therefore, the PAN fiber undergoes no recrystallization, even under fixed tension. However, the decreased mobility of the PAN molecular chains allows large-sized, highly oriented crystals that did not participate in the cyclization reaction to maintain their orientation.

Through WAXD analysis of T800, it is evident that carbon fibers with excellent mechanical properties exhibit high orientation and crystallinity. Therefore, during the temperature increase stage after the cyclization reaction, PAN fibers can be appropriately stretched to utilize PAN molecular chains’ better mobility, thereby slowing down crystal orientation reduction. By appropriately extending the retention temperature stage after the cyclization reaction, the PAN molecular chains can undergo complete cyclization reactions, allowing more PAN molecular chains to be converted into heat-resistant ladder structures to enhance the crystallinity of carbon fiber.

#### 3.3.2. Research on the Microporous Structure of PAN Fiber

**Figure 12:**
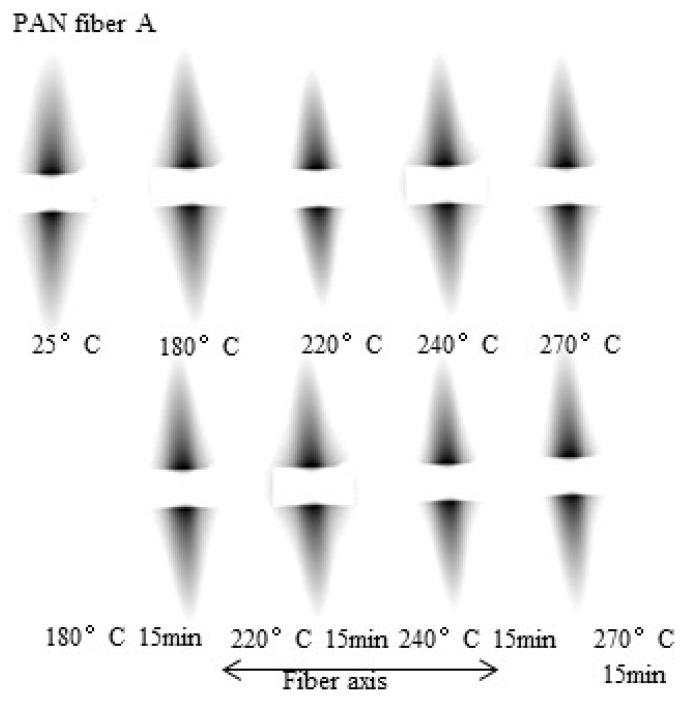
2D SAXS patterns of PAN fiber A during the pre-oxidation process.

Defective structures can significantly impact the mechanical properties of carbon fibers [34,35]. Therefore, in situ SAXS analysis was applied for the microporous structure of PAN fibers during both the temperature increase and isothermal retention stages of the pre-oxidation process to understand the evolutions in this structure. The two-dimensional scattering pattern of PAN fiber A is shown in Figure 12. The microporous scattering exhibited a diamond-shaped pattern throughout the pre-oxidation process, indicating that the micropores have a relatively elongated needle-like structure.

**Figure 13:**
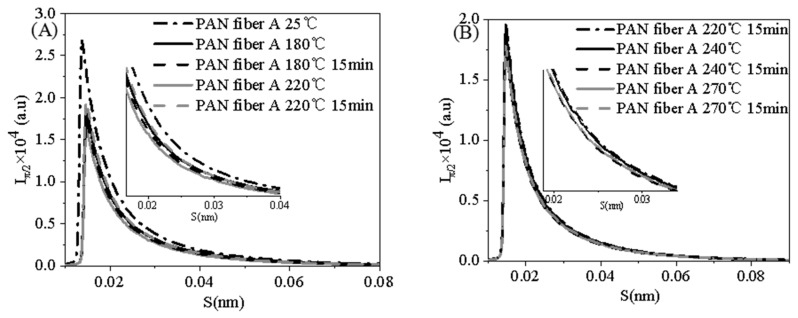

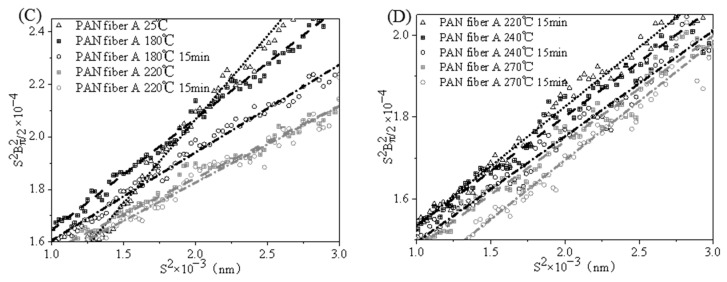
(**A**,**B**) Linear SAXS profiles of PAN fiber A during the pre-oxidation process, and (**C**,**D**) S_2_~$S^{2}B_{\pi /2}^{2}$ (S) plot of PAN fiber A during the pre-oxidation process.

The SAXS profiles of the PAN fiber with a 2° range perpendicular to the fiber axis during the pre-oxidation process are presented in Figure 13A,B. The micro-pore length (L) and orientation angle (B_eq_) were obtained by linear fitting of the S^2^~$S^{2}B_{\pi /2}^{2}$(S) plot, as shown in Figure 13C,D. The absence of a scattering peak in Figure 13A,B indicates that the PAN fiber A lacks periodic structure throughout the pre-oxidation process. The average chord length and relative micropore volume were obtained by taking the intensity values of medium and high angles into account using Equations (7) and (8). The micropore parameters of PAN fiber A were calculated from Figure 13, as shown in Table 6.

According to Table 6, during the temperature increase stage from 25 °C to 180 °C, the length of the micropores decreased while the average chord length increased. This suggests a reduction in the relative micropore volume and indicates the micropores’ shrinkage. The reduction in the relative micropore volume was due to the melting of small-sized crystals, which increased the volume of PAN fiber molecular chains. This also led to a decrease in the relative micropore volume. At the same time, the melting of crystals enhanced the mobility of PAN fiber molecular chains, causing a decrease in the orientation angle of the micropores under fixed tension and an increase in micropore orientations. However, during the other stages, there was minimal change in the length of the micropores compared with the temperature increase stage before cyclization. This can be attributed to the relatively well-preserved crystal structure of the PAN fibers, which effectively prevent micropore expansion based on WAXD data.

**Figure 14:**
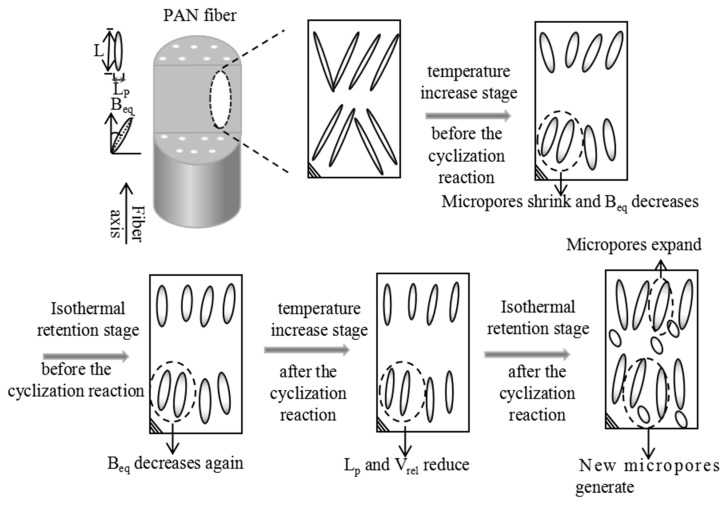
The micropore model of the PAN fiber during the pre-oxidation process.

Although there was no significant evolution in the length of the micropores, there were noticeable differences in the effects of the temperature increase and isothermal retention stages on the microporous structure. The evolution of the microporous structures of PAN fibers during the temperature increase and isothermal retention stages is illustrated in Figure 14.

1. Temperature increase stage before the cyclization reaction: The melting of crystals expands the volume of PAN fiber molecular chains, resulting in the shrinkage of micropores. Simultaneously, this melting process increases the molecular chain mobility of PAN fibers, leading to an enhanced orientation of micropores under fixed tension.
2. Isothermal retention stage before the cyclization reaction: The crystal structure is well maintained during this stage, leading to minimal evolution of micropores. Moreover, no cyclization reaction occurs in the PAN fibers, allowing the PAN chains to maintain a relatively high mobility. Under the influence of fixed tension, the orientation of micropores increases.
3. Temperature increase stage after the cyclization reaction: The average chord length (L_p_), the orientation angle of the micropores (B_eq_), and the relative micropore volume (V_rel_) decrease during this stage. This phenomenon can be attributed to the melting of crystals, which expands the PAN fiber molecular chains’ volume, reducing the average chord length and the relative micropore volume. Furthermore, only a few PAN molecular chains occur in the cyclization reactions, allowing the PAN molecular chains to maintain a high degree of mobility. Consequently, the orientation angle decreases, increasing the micropore orientation under fixed tension.
4. Isothermal retention stage after the cyclization reaction: The average chord length, the relative micropore volume, and the orientation angle of the micropores increase. This is attributed to the prominent cyclization reaction that occurs in this stage, leading to intermolecular or intramolecular cross-linking of the molecular chains in PAN fibers. As a result, the distance between PAN fiber molecular chains decreases [46], leading to an increase in the average chord length of the micropores. Additionally, the generation of new, small molecular gases after the cyclization reaction contributes to the increase in the relative micropore volume. Furthermore, the degree of mobility of PAN fiber molecular chains decreases after the cyclization reaction, weakening the influence of fixed tension on micropores and thus reducing the overall orientation of the micropores.

Based on the micropore data for T800, it is evident that carbon fibers with excellent mechanical properties exhibit relatively elongated micropore structures. However, the presence of crystals throughout the pre-oxidation process effectively hinders an increase in micropore length. Once these crystals disappear and the cyclization reaction reaches completion, the mobility of PAN molecular chain mobility decreases, thereby impeding alterations in the micropore structure. Consequently, appropriately adjusting the heating rate during the temperature increase stage is advisable to form a relatively elongated micropore structure in PAN fibers. This adjustment guarantees that the PAN fiber crystals entirely melt during the temperature increase stage, reducing the average chord length and ultimately resulting in fibers possessing elongated micropores.

## 4. Conclusions

This paper presents an in situ study of the pre-oxidation process of PAN fibers, which differs from previous research on the pre-oxidation of PAN fibers. This research analyzes the evolution of the microstructure of PAN fibers under two separate factors influencing the temperature increase and isothermal retention stages. As a result, the pre-oxidation process can be divided into four distinct stages, as shown below:

Temperature increase stage before the cyclization reactions: In this stage, only small-sized crystals melt, and the degree of crystal orientation increases under fixed tension. The melting of crystals results in the shrinkage of micropores.

Isothermal retention stage before the cyclization reaction: The crystal structure is well maintained during this stage, leading to minimal evolution of micropores. The orientation of crystals and micropores increases under fixed tension.

Temperature increase stage after the cyclization reactions: The crystal melts again, leading the average chord length and relative micropore volume to decrease. Additionally, only a few of the PAN molecular chains undergo cyclization reactions. As a result, recrystallization occurs.

Isothermal retention stage after the cyclization reaction: The PAN fiber crystal noticeably melts, with many PAN molecular chains undergoing the cyclization reaction, generating new, small molecular gases and decreasing the distance between PAN fiber molecular chains. This result leads to an increase in the average chord length of the micropores and the relative volume of the micropores. However, large-sized, highly oriented crystals do not participate in the cyclization reaction and maintain their orientation well.

Furthermore, through the analysis of the carbon fibers with different mechanical properties, it was observed that high-performance carbon fibers exhibit higher crystal orientation, increased crystallinity, and elongated micropore structures. Based on this observation, the following improvements are proposed for the pre-oxidation process of PAN fibers: 1. Slowing down the reduction of crystal orientation by appropriate stretching during the temperature increase stage after the cyclization reactions. 2. Extending the retention temperature stage after the cyclization reactions to enhance the crystallinity of the carbon fibers. 3. Adjusting the heating rate appropriately during the temperature increase stage to create a relatively elongated micropore structure in the PAN fibers.

## Figures and Tables

**Table 1:** Process parameters of PAN fibers used.

Sample	Coagulation Draft Ratio	Water Bath Draft Ratio	Vapor Bath Draft Ratio
PAN fiber A	2	2	2.2

**Table 2:** Process parameters and mechanical properties of carbon fibers ^a^.

	Carbon Fiber A	Toray T800	Retention Time (min)	Atmosphere
Pre-oxidation temperature (°C)	180–270	-	70	Air
low-temperature carbonization (°C)	900	-	1	N_2_
high-temperature carbonization (°C)	1500		1	N_2_
Tensile strength/GPa	4.9 GPa	5.9 GPa	-	-
Tensile modulus/GPa	230 GPa	294 GPa	-	-

^a^ carbon fiber A was prepared from PAN fiber A; the flow rate of N_2_ was 0.95 m^3^/min.

**Table 3:** Process parameters of pre-oxidation fibers ^a^ used.

Sample	Pre-Oxidation Temperature (°C)			
PAN fiber A	180	220	240	270

^a^ pre-oxidation fibers were prepared using PAN fiber A.

**Table 4:** Crystal parameters of the (002) crystal plane and micropore parameters ^a^ for T800 carbon fiber and carbon fiber A.

Sample	2θ (°)	Hermans Orientation	Crystal Size (nm)	L (nm)	B_eq_ (°)	L_p_(nm)	L/L_p_	V_rel_
T800	25.7	0.84	1.64	191.92	20.24	2.49	76.94	7.74
carbon fiber A	25.7	0.78	1.60	96.96	13.05	4.35	22.31	4.90

^a^ L = the length of the micropores; L_P_ = average chord length; L/L_P_ = aspect ratio; V_rel_ = the relative micropore volume; B_eq_ = orientation angle.

**Table 5:** Crystal parameters of the (100) crystal plane for PAN fiber A produced in the pre-oxidation process.

Sample	Crystal Size (nm)	Hermans Orientation	Temperature (°C)	Retention Time ^a^ (min)
PAN fiber A	8.97	0.59	25	-
PAN fiber A	12.77	0.64	180	-
PAN fiber A	13.18	0.72	180	15
PAN fiber A	14.74	0.65	220	-
PAN fiber A	14.18	0.68	220	15
PAN fiber A	13.94	0.64	240	-
PAN fiber A	11.29	0.77	240	15
PAN fiber A	10.04	0.63	270	-
PAN fiber A	5.66	0.70	270	15

^a^ Retention time: Once the temperature reaches the desired setting, maintain the temperature for 15 s before beginning the test.

**Table 6:** Micropore parameters ^a^ of PAN fiber A.

Sample	L (nm)	B_eq_(°)	L_p_ (nm)	L/L_p_	V_rel_	Temperature (°C)	Retention Time (min)
PAN fiber A	112.51	14.47	3.94	28.54	2.99	25	-
PAN fiber A	90.63	11.80	5.03	18.00	1.22	180	-
PAN fiber A	88.69	10.48	5.42	16.35	1.26	180	15
PAN fiber A	87.94	9.52	5.67	15.51	1.00	220	-
PAN fiber A	89.65	9.77	5.25	17.07	2.50	220	15
PAN fiber A	88.81	9.36	3.63	24.44	1.52	240	-
PAN fiber A	89.95	9.21	4.57	19.69	2.39	240	15
PAN fiber A	90.45	9.22	4.56	19.85	1.38	270	-
PAN fiber A	94.61	9.73	4.84	19.55	2.30	270	15

^a^ L = the length of the micropores; L_P_ = the average chord length; L/L_P_ = the aspect ratio; V_rel_ = the relative micropore volume; B_eq_ = the orientation angle of the micropores.

## Data Availability

The data presented in this study are not accessible for privacy reasons.
